# Extravascular distribution of factor IX—review of experimental and clinical evidence, and relevance for hemophilia B replacement therapy

**DOI:** 10.1016/j.rpth.2026.106862

**Published:** 2026-07-15

**Authors:** Cédric Hermans, Carmen Escuriola Ettingshausen, Michael Laffan, Davide Matino, Niamh O’Connell, Robert F. Sidonio, Rolf Ljung

**Affiliations:** 1 Division of Haematology, Saint-Luc University Hospital, Université catholique de Louvain, Brussels, Belgium; 2 Haemophilia Centre Rhein Main – HZRM, Frankfurt, Germany; 3 Centre for Haematology, Imperial College London, London, UK; 4 McMaster University, Hamilton, Canada; 5 National Coagulation Centre, St James’s Hospital, Dublin, Ireland; 6 Emory University and HOG Center for Bleeding and Clotting Disorders, Atlanta, Georgia, USA; 7 Lund University, Department of Clinical Sciences – Paediatrics, Lund, Sweden

**Keywords:** bleeding, extravascular distribution, factor IX, hemophilia B, replacement therapy, type IV collagen

## Abstract

Factor IX (FIX) is a relatively small molecule (55 kDa) that distributes physiologically beyond the plasma pool into the extravascular space. This contrasts with factor VIII, which is confined to the bloodstream as a consequence of its larger size (∼300 kDa) and binding to von Willebrand factor. The extravascular distribution of FIX may be significant in relation to factor replacement therapy in patients with hemophilia B. Given the increasing number of relevant publications over recent years, we critically reviewed and evaluated the experimental and clinical data relating to the extravascular distribution of FIX, considering both the native molecule and therapeutic products generated via biotechnological approaches. Endogenous FIX has an extensive distribution to tissues outside of plasma and is in dynamic equilibrium between plasma and the extravascular compartment. Approximately two-thirds of total FIX exists reversibly bound to type IV collagen in the vessel wall and extravascular space. This may produce an extravascular reservoir of FIX extending the plasma hemostatic role. Consequently, plasma FIX activity alone may not fully reflect the hemostatic potential of FIX prophylactic treatment. Extravascular FIX could be relevant for controlling and preventing both clinical and subclinical bleeds. When used as prophylactic treatment, extended half-life FIX products demonstrate similar annualized bleeding rates, but with a wide range of FIX trough levels and varying extents of extravascular distribution. Clinical implications of variation between FIX products remain to be fully understood; more knowledge is required. Further studies may help to address knowledge gaps, potentially informing treatment optimization.

## Essentials

• The bleeding disorder hemophilia B results from deficiency of coagulation factor IX (FIX).
• Endogenous FIX resides intravascularly but reaches other tissues, potentially aiding clotting.
• Used to prevent/stop bleeding, FIX products have different formulations and body distributions.
• Hence, FIX measured in the bloodstream may not fully reflect bleed protection.

## 1 Introduction

Hemophilia A and B result from pathogenic variants in the *F8* and *F9* genes causing deficiency or dysfunction of factor VIII (FVIII) and factor IX (FIX), respectively [1,2]. The *F9* gene is much smaller than *F8* (∼34 vs ∼180 kb) [1]. The molecular and clinical similarities and differences between the 2 conditions have been considered in detail previously [3]. Notably, hemophilia B occurs less often than hemophilia A, comprising between 15% and 20% of all hemophilia cases [4] and affecting ∼1 in 26,000 males [5]. Much of the understanding of hemophilia B has been extrapolated from hemophilia A [6]; however, differences in the biology of the disease and the FIX molecule [7,8] should be considered to facilitate optimal management of people with hemophilia B.

Compared with hemophilia A, a higher proportion of persons with hemophilia B have missense variants in the underlying gene [1] (particularly in those with severe disease, 58% vs 15% [3]), and more are cross-reactive material (CRM) positive (CRM+; ∼55% vs 5%) [7]. Missense variants in the *F9* gene often result in the production of FIX protein that is dysfunctional but detectable via immunologic assays, resulting in positive CRM status [9].

Exposure to the missing FVIII or FIX protein via administration of exogenous factor concentrates may result in the development of an immune reaction, producing antibodies that can neutralize replacement therapy (inhibitors) [10]; most inhibitors develop within the first 50 exposure days [11]. In hemophilia B, inhibitors develop almost exclusively in persons with the severe form of the disease, occurring in ∼10% of cases [12]. This is less frequent than for hemophilia A, in which the cumulative incidence of inhibitors is ∼30% in previously untreated patients [4]. Data from people with hemophilia B show that the development of inhibitors is associated with nonsense variants and large deletions resulting in absent protein (CRM negativity), rather than missense variants [12].

Although FVIII (procofactor) and FIX (zymogen) function in a 1:1 complex when activated, they circulate at different concentrations in plasma (approximately 0.4 nM and 90 nM, respectively [13]). As a relatively large molecule (∼300 kDa) [7], the intravascular residence of FVIII has been well established; FVIII binds to the large von Willebrand factor (VWF) multimer [[14], [15], [16]], limiting its distribution to primarily within the bloodstream. Native, unmodified FIX (55 kDa) is much smaller than FVIII [7]. As reviewed comprehensively elsewhere, it can distribute out from the plasma pool into the extravascular space, where it binds to type IV collagen [17]. The presence of FIX in both plasma and the extravascular space may be relevant with regard to the modalities of hemophilia B replacement therapy and its monitoring.

With an increasing number of relevant publications over recent years, and growing appreciation of the possible importance of extravascular FIX in clinical management [18,19], we aimed to critically review and evaluate published experimental and clinical data relating to the extravascular distribution of FIX, both the native molecule and therapeutic products generated via different biotechnological approaches, summarizing current knowledge and outlining knowledge gaps. Extended half-life (EHL) FIX products exhibit varying degrees of difference in their extravascular presence [20]. This increases interest in the importance of understanding the extravascular component and any implications of this in relation to product efficacy or clinical management.

## 2 Methods

This narrative review is based on searches performed to gather relevant information from the MEDLINE and Embase databases, as well as from gray literature. Interrogation of MEDLINE and Embase in October 2025 using pre-established search terms (Supplementary Table) retrieved a total of 476 articles (excluding duplicates) published since 1957. After screening the titles and abstracts of these, 7 reviewers from 7 countries who had scientific/clinical expertise in hemophilia B collated and critically interpreted information from selected articles. Current knowledge about the extravascular distribution of FIX was then summarized and elaborated to consider clinical and therapeutic implications. The interpretation used information from the selected articles obtained via the literature search supplemented by additional material as required (a total of 88 publications).

## 3 Results and Discussion

**Figure 1:**
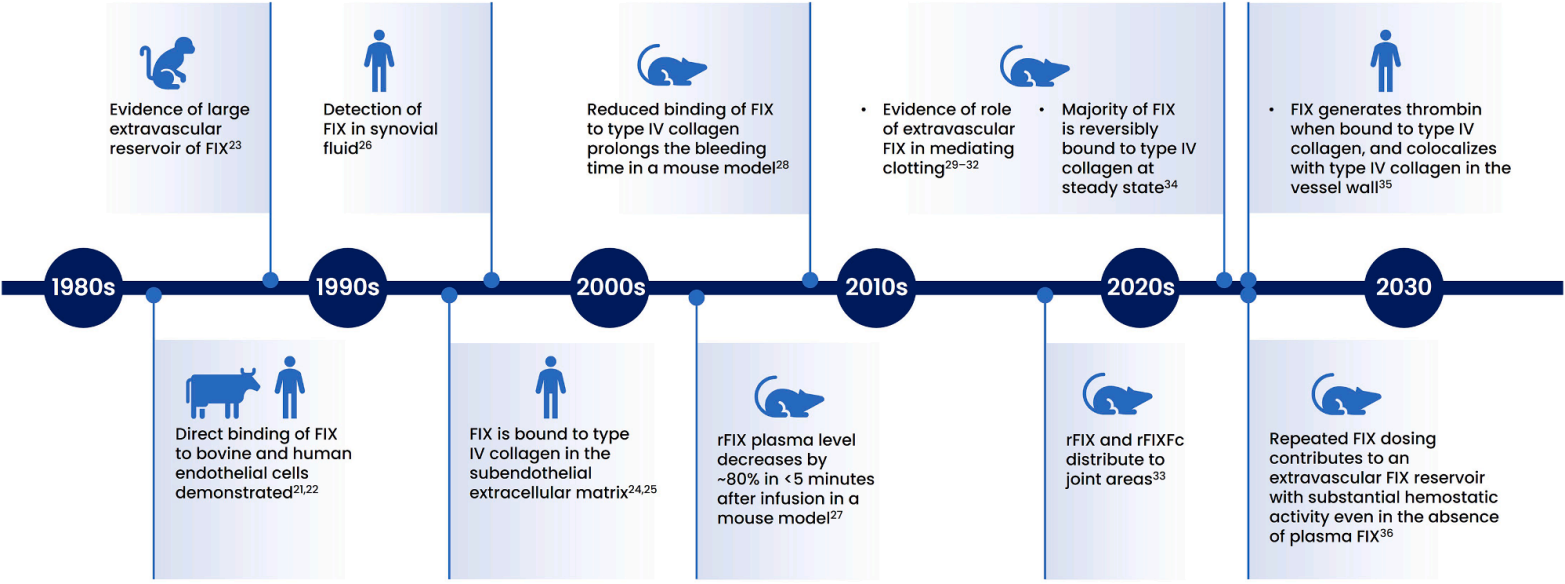
Key points relating to the extravascular distribution of FIX as reported over recent decades. FIX, factor IX; rFIX, recombinant factor IX; rFIXFc, recombinant factor IX Fc fusion protein.

Key points relating to the extravascular distribution of FIX as reported over the last 4 decades [[21], [22], [23], [24], [25], [26], [27], [28], [29], [30], [31], [32], [33], [34], [35], [36]] are summarized in Figure 1.

**Figure 2:**
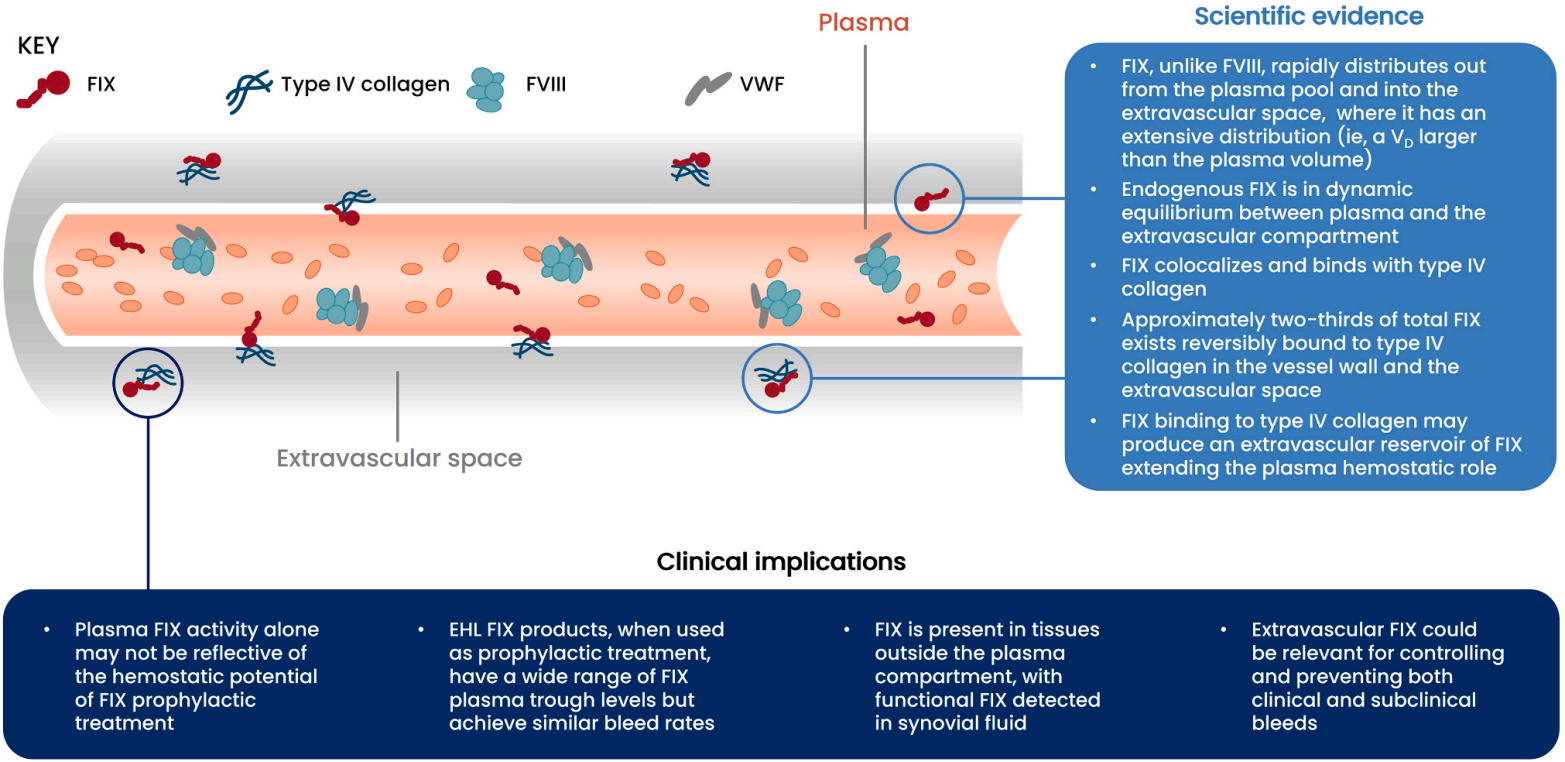
Key statements reflecting accumulated evidence relating to the extravascular distribution of FIX. EHL, extended half-life; FVIII, factor VIII; FIX, factor IX; V_D_, volume of distribution; VWF, von Willebrand factor. *Source:* image of blood vessel adapted from Astermark et al [6]. Copyright © The Authors, 2026. Reprinted by permission of Sage Publications.

Nine statements have been formulated to encapsulate current knowledge and understanding about the extravascular distribution of FIX (Figure 2). The statements are listed below, along with consideration of the evidence on which they are based, for which more detail is provided.

### **3.1** Scientific evidence relating to the extravascular distribution of FIX

#### 3.1.1 Statement/observation 1: FIX, unlike FVIII, rapidly distributes out from the plasma pool and into the extravascular space, where it has an extensive distribution (ie, a volume of distribution [V_D_] larger than the plasma volume)

FVIII in plasma exhibits rapid association with circulating VWF [20]. Considering V_D_ as a pharmacokinetic parameter quantifying distribution into both intra- and extravascular spaces, infused FVIII has a V_D_ close to the normal volume of blood plasma (40-41 mL/kg [20]). Reported mean V_D_ values range from 40 to ∼60 mL/kg across various FVIII products (plasma-derived, recombinant standard half-life [SHL], and EHL) [37,38].

In contrast, as demonstrated in previous work, within 5 minutes of being injected, 50% to 80% of infused unmodified FIX “disappears” from circulation [29]. For FIX, V_D_ values greater than the blood plasma volume are apparent [39], suggestive of FIX leaving the plasma and distributing to extravascular compartments [20]. Relevant pharmacokinetic information, collated from various sources, is shown in the Table [37,38,[40], [41], [42], [43], [44], [45], [46], [47]]. For plasma-derived FIX, V_D_ values ranging between 100 and 205 mL/kg have been reported [37,40,47], whereas for recombinant SHL FIX, a V_D_ value of 225 mL/kg has been described [41] (Table). The 3 available EHL FIX products, rFIX Fc fusion protein (rFIXFc), rFIX albumin fusion protein (rIX-FP), and glycopegylated rFIX (N9-GP), vary in their pharmacokinetic properties, with geometric mean V_D_ values ranging from 303 mL/kg for rFIXFc to 47 mL/kg for N9-GP [[42], [43], [44], [45]] (Table). Differences in pharmacokinetic parameters between plasma-derived and recombinant products may reflect variations in molecular structure and posttranslational characteristics, as well as methodological differences across pharmacokinetic studies.

**Table:** V_D_ and IR of various FIX products (based on separate studies).

Product	V_D_ during steady state (mL/kg)	IR (IU/dL per IU/kg)
pdFIX [37,40,47]	100-205^a^	1.23^b^
rFIX [41]	225^c^	0.74^c^
rFIXFc [42,43]	327^d^; 303^e^	1.02^d^; 0.92^e^
rIX-FP [44]	102^f^	1.30^f^
N9-GP [45]	47^g^	2.3^g^

For comparison, reported mean V_D_ values range from 40 to ∼60 mL/kg across various FVIII products (plasma-derived, recombinant standard half-life and extended half-life) [37,38], while a consensus empirical value of 2 IU/dL per IU/kg has been used to estimate recovery, and hence plasma levels, of FVIII [46].

FVIII, factor VIII; FIX, factor IX; N9-GP, glycopegylated recombinant factor IX; pdFIX, plasma-derived factor IX; rFIX, recombinant factor IX; rFIXFc, recombinant factor IX Fc fusion protein; rIX-FP, recombinant factor IX albumin fusion protein.

a Range of V_D_ values from various studies, including historical data (not all specified as during steady state) derived from less robust pharmacokinetic evaluations than for subsequently available products.

b Mean from 72 patients receiving treatment for spontaneous or traumatic bleeds or as surgical coverage.

c Mean based on a pharmacokinetic model using 30 subjects aged 18 to 60 years.

d Arithmetic mean based on a single 50 IU/kg dose in 22 subjects aged ≥18 years.

e Geometric mean based on a single 50 IU/kg dose in 22 subjects aged ≥19 years.

f Arithmetic mean based on a single 50 IU/kg dose in 47 subjects aged ≥18 years.

g Geometric mean based on a single 40 IU/kg dose in 6 subjects aged ≥18 years.

#### 3.1.2 Statement/observation 2: endogenous FIX is in dynamic equilibrium between plasma and the extravascular compartment

As previously described, patients receiving continuous infusion of FIX for surgery require decreasing amounts of FIX over time to maintain plasma levels of 100%, suggestive of binding sites being saturated and a substantial extravascular compartment [29]. However, extravascular FIX cannot be detected using conventional assays. Furthermore, CRM status may have an effect [9,47]. When product is administered, there may be more sites potentially available for extravascular binding in CRM− individuals. In those who are CRM+, administered FIX may have to compete with dysfunctional endogenous FIX for binding in the extravascular space. If this is the case, what are the implications for the treatment of CRM+ patients? In a post hoc analysis of data taken at a single timepoint from patients enrolled in the B-LONG study, CRM status did not influence the pharmacokinetics of rFIXFc and clinical efficacy, evaluated in terms of annualized bleeding rates (ABRs) and annualized joint bleeding rates, which were not significantly different between persons with hemophilia B caused by missense or non-missense variants [48]. However, CRM+ patients are not a homogeneous subpopulation but comprise individuals with a large spectrum of genetic variants, in whom the endogenous FIX molecules differ, resulting in variable amounts of inactive/dysfunctional FIX measurable in plasma. Clinical implications of the extravascular distribution of FIX are considered in more detail below.

Pharmacokinetic analyses show that FIX in the plasma and extravascular space is in dynamic equilibrium [20,34]. FIX product pharmacokinetic data provide further detail about this equilibrium. The pharmacokinetics of rFIXFc are best described by a 3-compartment model (as for plasma-derived FIX [49]), whereas N9-GP and rIX-FP can be described by 1- and 2-compartment models (as for FVIII) [[50], [51], [52], [53]]. A 3-compartment model may be indicative of a product with a high degree of extravascular distribution, and/or protein binding, returning to the plasma after initial clearance. This has implications for measured product recovery; lower *in vivo* recovery may be apparent when V_D_ is higher [20] (as discussed further in relation to Statement/observation 6, “Plasma FIX activity alone may not be reflective of the hemostatic potential of FIX prophylactic treatment”).

#### 3.1.3 Statement/observation 3: FIX colocalizes and binds with type IV collagen

Type IV collagen is a major component of cellular basement membranes [54]. Accumulated data from *in vitro* studies involving cultured endothelial cells have shown that human FIX binds to type IV collagen in the basement membrane via its γ-carboxyglutamic acid (Gla) domain [21,22,24,25,55]. In histologic evaluation of human liver sections stained for FIX and type IV collagen, the staining generally coincides in the subendothelial basement membrane [30]. Immunofluorescent staining has shown rFIX administered to hemophilia B mice exhibits spatial overlap with type IV collagen in the liver [32]. Additionally, other murine studies have observed extravascular localization of rFIX and type IV collagen in the same areas of various tissues [31,36]. One recent study demonstrated colocalization of FIX and type IV collagen in human synovial tissue sections and further confirmed binding of FIX to collagen via surface plasmon resonance binding assays [35].

Pharmacokinetic modeling based on clinical studies shows the total area under the curve of rFIXFc bound to type IV collagen in the extravascular space to be around 19 times higher than the area under the curve of rFIXFc in plasma [56].

#### 3.1.4 Statement/observation 4: approximately two-thirds of total FIX exists reversibly bound to type IV collagen in the vessel wall and extravascular space

Species-specific immunoassays showed that administration of bovine FIX to baboons resulted in an increase in plasma levels of baboon FIX, with a comparable decrease in levels of circulating bovine FIX (as measured via baboon and bovine FIX antigens in plasma), suggesting displacement of unmodified extravascular baboon FIX back into the bloodstream [23,29]. Subsequent pharmacokinetic studies have investigated this further, demonstrating the dynamic equilibrium between extravascular type IV collagen-bound FIX and FIX in the plasma [9,34].

**Figure 3:**
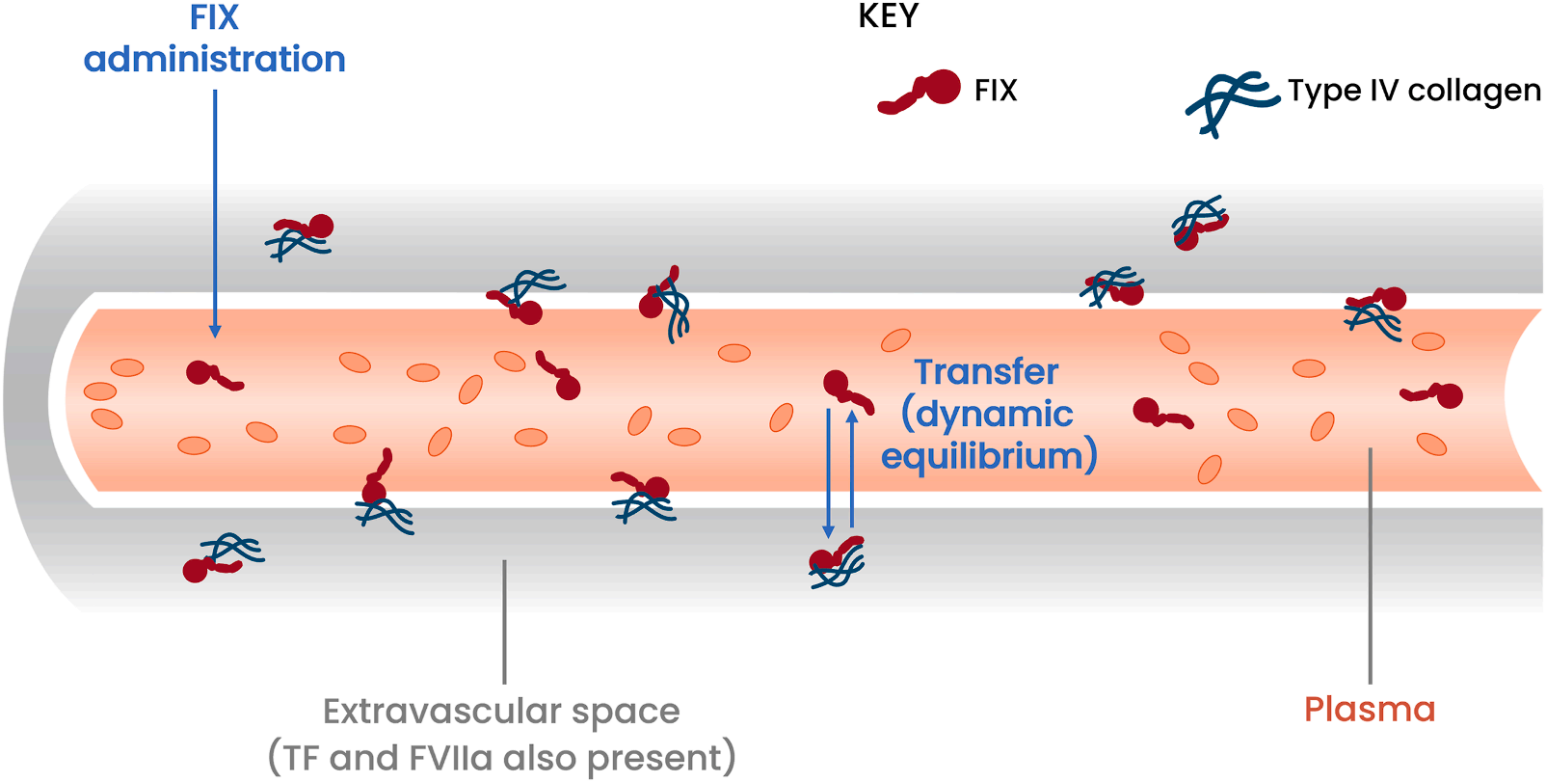
FIX replacement product distribution. As well as being present unbound in the plasma, administered FIX may bind to type IV collagen in the vessel wall and extravascular space, from where it is able to subsequently return to the plasma. In addition to collagen-bound FIX, the extracellular matrix contains other initiators of coagulation—TF and FVIIa. FVIIa, activated factor VII; FIX, factor IX; TF, tissue factor. *Source:* image of blood vessel adapted from Astermark et al. [6]. Copyright © The Authors, 2026. Reprinted by permission of Sage Publications.

Figure 3 [6] illustrates how administered FIX may be present unbound in the plasma while also being found bound to type IV collagen in the vessel wall and extravascular space, from where it is able to subsequently return to the plasma. In addition to collagen-bound FIX, as previously highlighted in this context, the extracellular space contains other initiators of coagulation, with both tissue factor and factor VII (FVII) surrounding vessels [30]. Proteolytic activation of FIX may subsequently occur [57]. It is possible that FIX may bind to activated FVII and tissue factor complexes in the perivascular space [58].

Recent data generated in murine models continue to provide more information, suggesting, for example, that after internal bleeding, extravascular coagulation may have a critical role in hemostatic plug formation [59]. In addition, protease domain exosites may regulate extravascular binding of FIX, but whether there are any additive or synergistic effects remains to be determined [60].

#### 3.1.5 Statement/observation 5: FIX binding to type IV collagen may produce an extravascular reservoir of FIX, extending the plasma hemostatic role

In hemophilia B mice injected with rFIX, although not detectable in plasma 3 days after administration, a hemostatic effect of extravascular FIX is evident [30,34]. Partial localized bleed protection is apparent 7 days after treatment when compared with untreated controls, as shown by results from saphenous vein bleeding assays. Bleeding challenge subsequently repeated on the contralateral vein confirmed these results, suggestive of an extravascular reservoir of FIX rather than undetectable but functional FIX circulating in the plasma [34]. Subsequent data from another saphenous vein hemophilia B mouse model have also shown extravascular FIX to provide bleed protection [36] (as discussed in relation to Statement/observation 9, “Extravascular FIX could be relevant for controlling and preventing both clinical and subclinical bleeds”).

In a subsequently reported study involving injection of rFIX into FIX knock-out mice, although FIX activity was below the detection limit (<1%) after 24 hours, after 5 days of moderate treadmill running (30 minutes daily), the bleeding score was significantly lower (*P* < .05) than that in a saline-treated control group [31].

In the FIX γ-carboxyglutamic acid-rich domain, replacement of lysine 5 with either alanine, FIX_K5A_, or arginine, FIX_K5R_, alters the affinity to type IV collagen [61]. By binding to type IV collagen, FIX may be sequestered in locations affected by bleeding, with consequences for bleed protection. As reported in an earlier review, FIX_K5A_ has increased *in vitro* activity (up to 2-fold) compared with wild-type FIX [17] but reduced binding to type IV collagen [27]. Knock-in mice genetically modified to express FIX_K5A_ exhibit a bleeding diathesis despite having FIX activity in plasma higher than that of wild-type [28]. FIX_K5R_ has increased affinity for type IV collagen, and hemophilia B mice dosed with FIX_K5R_ exhibited hemostatic protection 7 days after treatment, despite having FIX plasma levels of <1% after 3 days. Additional *in vitro* data have shown that FIX bound to type IV collagen remains available for normal thrombin generation [35].

### 3.2 Clinical implications of the extravascular distribution of FIX

#### 3.2.1 Statement/observation 6: plasma FIX activity alone may not be reflective of the hemostatic potential of FIX prophylactic treatment

Although findings show hemostatic benefits of extravascular FIX [30,34,36], FIX assays only detect FIX activity in plasma. As evidenced in murine studies [30,31,34], these plasma levels may underestimate the true hemostatic state.

The pharmacokinetic properties of rFIX differ from those of plasma-derived FIX [37], posttranslational modification affects product characteristics [36], and across the range of EHL FIX products, half-life extension technology may impact distribution to the extravascular space. As suggested by V_D_ values for rFIXFc, rIX-FP, and N9-GP (Table), rFIXFc would be expected to exhibit substantial distribution to extravascular space, and less so for rIX-FP, while N9-GP may be limited more to plasma. In rFIXFc, FIX is covalently fused to the human immunoglobulin G1 Fc domain [62]. This extends product half-life; it delays lysosomal degradation as a consequence of binding to neonatal Fc receptors, which exhibit lifelong expression on several types of cells, including those comprising the vascular endothelial lining [63]. rFIXFc can subsequently recycle back into the circulation [63]. In rIX-FP, fusion of FIX to albumin [44] also results in half-life extension via neonatal Fc receptor-mediated recycling [64]. With albumin fusion, the molecular weight of rIX-FP is ∼125 kDa [65], and it has been postulated that the size of this molecule may limit its access to the extravascular space [66]. N9-GP comprises a FIX molecule in which a 40 kDa polyethylene glycol (PEG) moiety is attached to the FIX activation peptide, which is cleaved upon FIX activation [45]. As a consequence of PEGylation, the efficiency of several physiological elimination processes is impaired and molecules can persist in the bloodstream for longer than would otherwise be possible, resulting in half-life extension [67]. *In vitro* data show N9-GP to exhibit reduced endothelial cell binding compared with rFIX [68]. It is possible that steric effects may inhibit potential for collagen binding or extravasation.

With increased distribution to the extravascular space, monitoring product plasma FIX activity levels may not provide a full indication of the amount of FIX available for hemostasis.

#### 3.2.2 Statement/observation 7: EHL FIX products, when used as prophylactic treatment, have a wide range of plasma trough levels but achieve similar bleed rates

In the absence of direct, head-to-head comparisons, matching-adjusted indirect comparison (MAIC) [69,70] can be used to assess the efficacy of EHL FIX products. In MAIC analyses, individual patient data from one study are matched to the aggregated (summarized) data in a comparator trial by weighting patient-level data to balance between-trial differences in the baseline characteristics of the 2 populations. This is intended to account for the differences between the patient populations in the 2 trials. The efficacy outcome for the reweighted population can then be recalculated to facilitate comparison between the 2 studies. As a validated technology recognized by bodies responsible for health assessment, this has been increasingly used over recent years [71]. However, it is important to understand the limitations of such analyses; not all trials can be compared, the analyses are sensitive to the study design, and critical evaluation of the results is appropriate [71]. Furthermore, results from different MAIC analyses are not directly comparable due to methodological considerations.

MAIC has shown no significant differences in the efficacy of bleed prevention for pooled weekly and interval-adjusted dosing with rFIXFc (targeting FIX levels of 1-3 IU/dL) compared with N9-GP 40 IU/kg once weekly (observed mean FIX trough levels of 27.3 IU/dL) [72]. This analysis evaluated results from the pivotal phase 3 B-LONG [73] and PARADIGM-2 [74] trials, for rFIXFc and N9-GP, respectively, taking into account baseline characteristics of age, weight, ethnicity, receipt of prior prophylaxis, and presence of target joints. Estimated ABRs of 2.59 for rFIXFc and 2.51 for N9-GP (incidence rate ratio [IRR], 1.03; 95% CI, 0.56-1.89) for overall bleeding events, as well as 1.34 vs 1.22 (IRR, 1.10; 95% CI, 0.42-2.91) and 1.13 vs 1.29 (IRR, 0.88; 95% CI, 0.47-1.63), for spontaneous and traumatic bleeds, respectively, have been described [72].

Another MAIC [75] evaluated results for rFIXFc (targeting FIX levels of 1-3 IU/dL) and rIX-FP (observed mean FIX trough levels of 20 IU/dL) using data from the B-LONG [73] and phase 3 PROLONG-9FP [76] trials, respectively, according to prior treatment regimen at study entry. Patients’ baseline characteristics were matched for age, weight, ethnicity, and prior bleeding frequency. Again, no significant differences in the efficacy of bleed prevention were apparent. Estimated mean ABRs were 1.87 for rFIXFc and 1.58 for rIX-FP (IRR, 1.18; 95% CI, 0.67-2.10) in those who had received prior prophylaxis and 2.25 vs 2.22 (IRR, 1.01; 95% CI, 0.40-2.57) for patients with prior on-demand treatment [75].

An additional MAIC evaluation of rFIXFc and rIX-FP data from the B-LONG and PROLONG-9FP trials showed no significant difference between these treatments for ABRs, 3.12 and 2.35, respectively (rate ratio [RR], 0.75; 95% CI, 0.32-1.75; *P* = .51) and annualized joint bleeding rates, 2.26 vs 1.84 (RR, 0.82; 95% CI, 0.37-1.82; *P* = .62) [77], although decreases in favor of rIX-FP were reported in terms of the annualized spontaneous bleeding rate and the percentages of patients with zero bleeds overall, zero joint bleeds, and zero spontaneous bleeds. However, this analysis only included participants receiving weekly prophylaxis, and the adjustment for baseline characteristics (age, body mass index, disease severity, and prior FIX regimen) did not account for the broader inclusion criteria in the B-LONG trial. For example, there was no adjustment for the number of prior bleeds. As more patients who previously received on-demand treatment were enrolled in the B-LONG trial, this study may have included patients with more advanced arthropathy, possibly impacting subsequent prophylactic outcomes.

Other data have also suggested that the relationship between FIX plasma trough levels and efficacy is not straightforward. A survey to evaluate real-world performance of EHL FIX products in patients with severe hemophilia B treated across 6 centers in the United States and Canada showed differential responses between the treatments, with unexpected or poorly controlled bleeding in those receiving rIX-FP, despite some achieving FIX plasma trough levels >10% [78]; this highlights the value of additional understanding relating to the characteristics of individual patients and the extravascular presence of FIX [79].

Additional single-center data have described 3 of 25 rIX-FP-treated patients who experienced unexpectedly poor bleed control while receiving rIX-FP 60 to 65 IU/kg once every 14 days, none of whom had active inhibitors [66]. The authors concluded that clinicians who care for patients with hemophilia B receiving rIX-FP should be alert to unexpected breakthrough bleeding and speculated that the poor therapeutic response in some individuals may be related to the relatively low V_D_ of rIX-FP, with the albumin fusion molecule having limited access to the extravascular space [66]. Subsequent real-world data have not found higher ABRs with rIX-FP than other EHL products [80,81].

Previous clinical data generated during SHL FIX prophylaxis showed no strong association between FIX trough levels and ABR [82] and no apparent pattern between time of infusion and spontaneous bleeding events [83]. Recent modeling data that show considerable differences in the pharmacokinetic profiles between different EHL FIX products suggest that high or low plasma levels do not necessarily equate with improved or reduced bleed protection, respectively [84]. Virtual simulations with rIX-FP have shown bleeds may occur in treated patients with trough FIX levels >20 IU/dL [85].

#### 3.2.3 Statement/observation 8: FIX is present in tissues outside the plasma compartment, with functional FIX detected in synovial fluid

Assay of synovial fluid aspirated from persons without hemophilia who have osteoarthritis has shown FIX and other smaller coagulant proteins, factor XI and prothrombin, to be present in relatively high concentrations (approximately 10, 31, and 21% of the activity in normal pooled reference plasma, respectively), whereas concentrations of the larger coagulant proteins, factor V, FVIII, and VWF, are minimal or absent (activities <1% of those in reference plasma) [26]. However, thrombin can be generated in synovial fluid [26], demonstrating the functionality of synovial FIX.

In another study involving hemophilia B mice, immunohistochemical staining showed plasma-derived FIX to be present in some chondrocytes near the synovial membrane and on some synoviocytes of knee joints after supraclinical dosing [86]. Also, in hemophilia B mice, single-photon emission computed tomography imaging demonstrated that after injection, rFIX and rFIXFc distribute to tissues outside the plasma compartment, with localization to joint areas and muscle persisting at 5 half-lives post dosing [33,87]. However, recombinant glycopegylated FIX (generated by following published methods applied to rFIX and identical to N9-GP) showed less distribution with almost no localization to joint areas [87].

#### 3.2.4 Statement/observation 9: extravascular FIX could be relevant for controlling and preventing both clinical and subclinical bleeds

In hemophilia B mice treated with SHL FIX or rFIXFc, immunohistochemical results show SHL FIX and rFIXFc localization in the muscles, synovial membrane and knee joints of hind paws, with collagen binding [36]. Seven days after last dosing, when no FIX was detectable in the plasma, saphenous vein bleeding assays showed the extravascular procoagulant activity of FIX to be significantly higher after 5 days of prophylaxis than with a single dose [36]. This demonstrates that the FIX reservoir can be fed by regular prophylactic dosing. Extrapolating these data to the clinical environment may suggest that when managing individuals with hemophilia B, extravascular FIX could be relevant for prophylaxis. Perivascular stores of FIX might help to control microbleeds [36], although more research is needed. Indeed, the possibility of extravascular FIX helping to control microbleeds has also been postulated previously [34,88]. In addition to microbleeds, relatively large bleeds can also go undetected and are then by definition “subclinical”; we propose that the hemostatic effects of extravascular FIX could be relevant in this wider context.

Although the extravascular presence of FIX may confer hemostatic benefit, it is relevant to distinguish between routine prophylaxis, in which the extravascular space may be saturated (with the possibility of perivascular stores of FIX controlling microbleeds [36], as mentioned above), and coverage of invasive surgical procedures, where high concentrations of FIX in the vascular compartment are likely very important.

## 4 Concluding Remarks

Experimental and clinical evidence suggests FIX has a role in hemostasis that is not limited to its intravascular presence. The extravascular distribution of FIX is important and may have benefits for prophylaxis. Although the extravascular transfer of FIX is evident from pharmacokinetic data and *in vivo* binding to collagen, at present, it is not possible to directly measure FIX in extravascular tissues; work is underway to develop models to measure extravascular FIX [56].

The clinical implications of the variations between FIX products remain to be fully understood; more knowledge is required to build on the existing evidence. Indeed, further studies may help to address gaps in knowledge, potentially informing further optimization of the management of persons with hemophilia B. For example, CRM status may affect FIX recovery and prophylactic efficacy, and although CRM+ patients may exhibit higher FIX recovery, this may not correspond to improved outcomes [34]. In the future, as now, product choice should continue to be a result of shared decision making between healthcare providers and their patients. This should consider the contribution of both plasma and extravascular FIX to hemostasis. Notably, as FIX activity in plasma may not fully reflect overall hemostatic protection in the same way as FVIII plasma activity, clinical information about bleeding, including joint examination (physical or via imaging), is particularly important for optimizing treatment.

Awareness of the properties of FIX, including the differences from FVIII in relation to replacement therapy, should be increased within the community. Summarizing evidence of the extravascular distribution of FIX published throughout the decades, this article is intended to contribute accordingly. Knowledge has advanced over time, but more information is clearly required to further understand the biology of FIX and the characteristics of different FIX products to ensure the most effective use of factor replacement therapy in those with hemophilia B.
