# Supplementary material for: Extravascular distribution of factor IX—review of experimental and clinical evidence, and relevance for hemophilia B replacement therapy
Source: Res Pract Thromb Haemost. 2026 Jul 15;10(5):106862. doi: 10.1016/j.rpth.2026.106862 (PMC13476773; doi:10.1016/j.rpth.2026.106862)
Supplement: Supplementary file 1 [file mmc1.docx]

**Supplementary material**

**Supplementary Table 1.** Literature search terms (MEDLINE and Embase databases interrogated).

| Set | Searched for | Results |
| --- | --- | --- |
| S1 | MJEMB.EXACT.EXPLODE("blood clotting factor 9") OR MJEMB.EXACT.EXPLODE("recombinant blood clotting factor 9") OR MJEMB.EXACT.EXPLODE("blood clotting factor 9  concentrate") OR MJMESH.EXACT.EXPLODE("Factor IX") | 8075 |
| S2 | ti,ab((SHL OR "standard half life" OR "standard halflife" OR pd OR "plasma derived") NEAR/3 ("Factor 9" OR "Factor IX" OR "F9" OR "FIX")) | 391 |
| S3 | ti,ab(((EHL OR longacting or "long acting" OR "long lasting" OR "fc fusion" OR "albumin fusion" OR "fusion protein" OR glycopegylated OR pegylated OR PEG or "polyethylene glycol") NEAR/3 ("factor 9" OR "f9" OR "FIX"))) OR ti,ab((exten* OR prolong*) NEAR/3 ("half life" OR halflife) NEAR/3 ("Factor 9" OR "Factor IX" OR "F9" OR "FIX")) | 499 |
| S4 | ti,ab,tn("Factor IX" OR "Factors IX" OR "F IX" OR "FIX product" OR "FIX products" OR "FIX concentrate*" OR "FIX blood clotting" OR "FIX blood coagulation" OR "FIX coagulation" OR "FIX Padua" OR "Christmas factor" OR "recombinant FIX" OR "r FIX" OR "rFIX" OR "pd FIX" OR "plasma derived FIX" OR Rixubis OR Benefix OR Alphanine OR "alpha nine" OR Immunine OR Octanine OR Ixinity OR Rebinyn OR Refixia OR Idelvion OR Alprolix OR "nonacog alfa" OR "nonacog gamma" OR "nonacog beta" OR "dalcinonacog alfa" OR "nonacog beta pegol" OR "albutrepenonacog alfa" OR "eftrenonacog alfa" OR "N9-GP" OR "rFIX GP" OR "rIX-FP" OR "rFIX-Fc" OR "rFIXFc") | 14051 |
| S5 | S1 OR S2 OR S3 OR S4 | 16431 |
| S6 | ti,ab,su(crm OR "cross-reactive material") | 13387 |
| S7 | ti,ab,su,if("vascular wall" OR subendothel* OR "subendothel*" OR "endothel* barrier" OR "endothel* wall" OR "intercellular matrix" OR "tissue distribut*" OR "tissue  concentration*" OR "tissue level*" OR "tissue locali*" OR extravas* OR "extra vas*" OR "extracellular matrix" OR "basement membrane*" OR "basal membrane*" OR intima OR laminin OR "collagen 4" OR "collagen iv" OR "collagen type 4" OR "collagen type IV" OR "type 4 collagen" OR "type IV collagen" OR "col 4" OR "col IV" OR "collagen 1" OR "collagen I" OR "collagen type 1" OR "collagen type I" OR "type 1 collagen" OR "type I collagen" OR "col 1" OR "col I" OR "subendothelial collagen" OR "basal lamina") | 1164620 |
| S8 | ti,ab,su,if((bound OR binding OR binds OR bind OR interact* OR affinity OR distribut* OR biodistribut* OR "bio distr*" OR transport*) NEAR/6 (collagen OR endothel*)) | 99115 |
| S9 | (S6 OR S7 OR S8) AND S5 | 364 |
| S10 | ti,ab(((((SHL OR "standard half life" OR "standard halflife" OR pd OR "plasma derived") NEAR/3 ("Factor 9" OR "Factor IX"OR "F9" OR "FIX")) OR (((EHL OR longacting or "long acting" OR "long lasting" OR "fc fusion" OR "albumin fusion" OR "fusion protein" OR glycopegylated OR pegylated OR PEG or "polyethylene glycol") NEAR/3 ("Factor 9" OR "F9" OR "FIX"))) OR ((exten* OR prolong*) NEAR/3 ("half life" OR halflife) NEAR/3 ("Factor 9" OR "Factor IX" OR "F9" OR  "FIX")) OR ("Factor IX" OR "Factors IX" OR "F IX" OR "FIX product" OR "FIX products" OR "FIX concentrate*" OR "FIX blood clotting" OR "FIX blood coagulation" OR "FIX coagulation" OR "FIX Padua" OR "Christmas factor" OR  "recombinant FIX" OR "r FIX" OR "rFIX" OR "pd FIX" OR "plasma derived FIX" OR Rixubis OR Benefix OR Alphanine OR "alpha nine" OR Immunine OR Octanine OR Ixinity OR Rebinyn OR Refixia OR Idelvion OR Alprolix OR "nonacog alfa" OR "nonacog gamma" OR "nonacog beta" OR "dalcinonacog alfa" OR "nonacog beta pegol" OR "albutrepenonacog alfa" OR "eftrenonacog alfa" OR "N9-GP" OR "rFIX GP" OR "rIX-FP" OR "rFIX-Fc" OR "rFIXFc")))) | 13796 |
| S11 | ti,ab(distribut* OR biodistribut* OR "bio distr*" OR transport* OR accumulat* OR subendothel* OR endothel* OR "subendothel*" OR "extracellular matrix" OR "intra cellular" OR intracellular OR storage OR traffic* OR laminin OR intercellular OR "inter cellular" OR ((level* OR concentration OR locali*) NEAR/2 ("tissue*"))) | 9000939 |
| S12 | S10 NEAR/10 S11 | 212 |
| S13 | S9 OR S12 | 476* |

*Duplicates removed from the search and from the result count.
